# Serum Alpha-Fetoprotein-Tumor Size Ratio as a Prognostic Marker After Hepatic Resection for Primary Hepatocellular Carcinoma: Propensity Score Matched Retrospective Cohort Study

**DOI:** 10.2196/64929

**Published:** 2025-08-26

**Authors:** Shutian Mo, Yongfei He, Tianyi Liang, Guangzhi Zhu, Hao Su, Chuangye Han, Tao Peng

**Affiliations:** 1 Department of Hepatobiliary Surgery, The First Affiliated Hospital of Guangxi Medical University, No 6 Shuangyong Road, Guangxi Zhuang Autonomous Region, Nanning, 530021, People’s Republic of China, 86 13978691700; 2 Guangxi Key Laboratory of Enhanced Recovery After Surgery for Gastrointestinal Cancer, Guangxi Zhuang Autonomous Region, Nannning, People’s Republic of China

**Keywords:** hepatocellular carcinoma, alpha-fetoprotein, hepatectomy, prognosis, nomograms, propensity score

## Abstract

**Background:**

Patients with hepatocellular carcinoma (HCC) exhibit a high rate of recurrence and poor prognosis after surgery, and effective prognostic indicators and stratification strategies are currently lacking. Hence, this study proposes new prognostic markers to provide a theoretical basis for patients with HCC.

**Objective:**

We aim to build and evaluate a model estimating the effect of alpha-fetoprotein-tumor size ratio (ATR) on the prognosis of patients undergoing hepatectomy for HCC.

**Methods:**

We retrospectively reviewed hospital records to identify patients who underwent hepatectomy for HCC at the First Affiliated Hospital of Guangxi Medical University from January 2013 to December 2018. Outcomes (recurrence events and mortality) not available in the outpatient medical records were determined through telephone interviews until February 2022. The optimal cutoff value was determined using X-tile (Yale School of Medicine). Independent risk factors for prognosis were investigated by Cox regression modeling, and between-group differences were reduced through propensity score matching. A predictive model for HCC prognosis was constructed using a nomogram, and the predictive performance of the model was evaluated using the C-index.

**Results:**

Of the 1628 eligible patients, 1204 patients were included in the analysis. Patients were stratified into low, medium, and high ATR groups with X-tile. Before propensity score matching, ATR was identified as an independent risk factor for overall survival (low vs medium: HR 1.41, 95% CI 1.03‐1.94; *P*=.03; medium versus high: HR 1.59, 95% CI 1.02‐2.47; *P*=.04) and relapse-free survival (low vs medium: HR 1.33, 95% CI 1.03‐1.70; *P*=.03; medium versus high: HR 2.10, 95% CI 1.40‐3.15; *P<.*001) of patients with HCC following hepatectomy. A nomogram incorporating ATR, China Clinic Liver Cancer staging, bleeding, and postoperative transcatheter arterial chemoembolization was developed to predict moderate predictive efficacy for overall survival (C-index: 0.73) and relapse-free survival (C-index: 0.73). ATR was found to be associated with microvascular, macroinvasion, and poor tumor differentiation.

**Conclusions:**

ATR is an independent prognostic risk factor in patients with HCC after hepatectomy and is associated with microvascular, macroinvasion, and poor tumor differentiation.

## Introduction

Primary liver cancer is among the leading causes of tumor-related deaths worldwide. In 2022, liver cancer registered the highest incidence and mortality rates, representing a considerable public health challenge. Hepatocellular carcinoma (HCC) accounts for over 75% of primary liver cancer cases [1,2]. Although mortality rates from other cancers are declining, HCC continues to exhibit increasing cancer-related mortality. The primary treatment modality for HCC encompasses a combination of surgical interventions and interventional, targeted, immunological, and herbal treatments. Hepatectomy remains the most prominent curative approach for patients with HCC [3]. The high malignancy and heterogeneity of HCC, coupled with the lack of well-defined stratification strategies and postoperative follow-up protocols, contribute to the poor prognosis of HCC. The 5-year survival rate remains only 15%, and the rate of early recurrence reaches approximately 70% within 5 years [3-5]. In this regard, identifying reliable prognostic markers is essential to identify high-risk patients, and informing the development of effective stratification and postoperative follow-up strategies is urgently needed.

Serum alpha-fetoprotein (AFP) is mainly produced by the liver and yolk sac during the fetal and neonatal periods, and its levels decline rapidly following birth (<5 ng/mL) [6]. AFP is an important marker for HCC diagnosis, and more than 70% of patients are AFP positive [7]. AFP serves not only as a diagnostic biomarker for HCC but also as an important indicator of its prognosis [8]. Tumor size is also an important prognostic factor for patients and should not be overlooked [9]. It has long been considered an important determinant of tumor staging and plays an important role in widely used staging systems, such as in the Milan criteria, Barcelona Clinic Liver Cancer (BCLC) staging system, and China Clinic Liver Cancer (CNLC) staging system [10,11]. AFP is secreted by tumor cells and is positively correlated with tumor size. Given this relationship, AFP and tumor size may exert a combined effect on HCC prognosis, warranting further investigation into their joint prognostic value.

AFP levels reflect the degree of HCC malignancy to some extent [12]. We speculate that when the tumor size is comparable, elevated AFP levels indicate a high degree of tumor malignancy and poor prognosis. This study focuses on the effect of the AFP-tumor size ratio (ATR) on the prognosis of patients with HCC and investigates the correlation between the ratio and the degree of tumor malignancy.

## Methods

### Participants

We retrospectively reviewed the records of patients who underwent hepatectomy for HCC at the First Affiliated Hospital of Guangxi Medical University from January 2013 to December 2018. Clinical data were obtained from the hospital’s database. Patients with missing postoperative follow-up data or other essential clinical information were excluded. The inclusion criteria were as follows (outlined in the flowchart): (1) pathological diagnosis of HCC, (2) complete postoperative follow-up and pathological information, and (3) hepatectomy as the initial treatment upon HCC diagnosis. The exclusion criteria were as follows: (1) comorbidities with other malignant tumors, (2) postoperative death within 30 days, (3) loss to postoperative follow-up, (4) diagnosis of recurrent liver tumor, and (5) history of previous hepatectomy.

### Surgical Approach

The First Affiliated Hospital of Guangxi Medical University is one of the largest hospitals in Guangxi Zhuang Autonomous Region of China, with approximately 2750 beds. The hospital conducts 70,000‐80,000 surgeries every year, including 400‐1000 liver resections. All patients undergo preoperative evaluation to confirm the absence of contraindications to surgery, cardiac function, pulmonary function, blood routine, liver function, and renal function. Postoperative residual liver volume and function are assessed on the basis of 15-minute indocyanine green retention rate and preoperative liver volumetry data. The surgical plan of all patients is determined through multidisciplinary or intradepartmental expert discussions. All 10 surgeons in the surgical group are highly experienced. Intraoperative hepatic tumor resection was performed using the Pringle maneuver, and postoperative abdominal lavage with indwelling drains was routinely conducted.

### Data Collection

Researchers (SM, TL, and YH) retrospectively collected baseline information in June 2021, including gender, age, BMI, history of smoking, alcohol consumption, hypertension, and diabetes mellitus. Preoperative liver function assessments included the presence of cirrhosis, Child-Pugh classification, and hepatitis status. Tumor-related information comprised tumor size, preoperative AFP level, number of tumors, macrovascular invasion, microvascular invasion (MVI), and degree of tumor differentiation. Tumor staging status was recorded using the BCLC and CNLC staging systems. Surgery-related parameters included surgery time, surgical bleeding, surgical approach, radical resection, major hepatectomy, and postoperative transcatheter arterial chemoembolization (TACE) treatment. Data were missing for 122 patients.

### Postoperative Follow-Up of Patients

Patients were followed up every 3 months within the first year following surgery and every 6 months thereafter for imaging, liver function tests, and AFP measurements. The primary outcome indicators were status and duration of tumor recurrence and overall survival (OS). Postoperative follow-up evaluations adhered to institutional clinical practices and were performed at 1, 3, 6, 12, and 18 months postoperatively and every 12 months thereafter until February 2022. These evaluations involved monitoring recurrence events, time to recurrence, OS, and mortality. Postoperative survival statuses were routinely updated in our cancer database annually through telephone interviews or by reviewing outpatient medical records. SM was responsible for patient postoperative follow-up, and for patients whose prognostic data were available in the hospital database, we obtained the patients’ prognostic data from the hospital database. If prognostic data were not available in the hospital database, we obtained patient prognostic data by telephone interview. Missing data in the database, wrong phone numbers, refusal to interview, and failure to connect after calling up at least three times were considered lost to postoperative follow-up. Finally, 302 patients were lost to postoperative follow-up.

### Nomogram

We used R-project with the “rms” and “survival” packages to construct prediction models for OS and relapse-free survival (RFS), incorporating independent risk factors for OS and RFS into the models. The predictive performance of the model was assessed using the C-index, and the accuracy of the model was evaluated using correction curves.

### Statistical Analysis

Continuous variables were expressed as mean (SD) or median (IQR) and statistically analyzed using a 2-tailed *t* test or Wilcoxon test. Categorical data were statistically analyzed using continuity correction or Fisher exact test. RFS and OS were calculated using the Kaplan-Meier method and compared using the log-rank method. X-tile (Yale School of Medicine) software was used to determine the optimal cutoff value [13]. Cox proportional risk models were used to determine independent risk factors for OS and RFS. Factors with *P* <.05 in the univariate analyses were included in the subsequent multivariate analyses. Hazard ratios (HRs) and 95% CIs were calculated for each variable in the models. Propensity score matching (PSM) using the greedy nearest neighbor method was performed to match covariates to minimize potential selection bias. Variables that showed statistically significant differences (*P*<.05) in the baseline data of the patients (Table 1) were included in the subsequent PSM analysis. The following variables were included: gender, BMI, diabetes, cirrhosis, tumor size, tumor number, macroinvasion, BCLC, CNLC, bleeding, radical resection, major resection, MVI, pathological grade, and postoperative follow-up TACE. A *P* value of <.05 indicated statistical significance.

**Table 1.** Patient’s baseline data.

Variables	Total (n=1204), n (%)	Low (n=499), n (%)	Medium (n=572), n (%)	High (n=133), n (%)	*P* value
Sex					.007
Female	187 (16)	58 (12)	104 (18)	25 (19)	
Male	1017 (84)	441 (88)	468 (82)	108 (81)	
Age (years)					.31
<60	963 (80)	389 (78)	464 (81)	110 (83)	
≥60	241 (20)	110 (22)	108 (19)	23 (17)	
BMI					<.001
<24	791 (66)	300 (60)	389 (68)	102 (77)	
≥24	413 (34)	199 (40)	183 (32)	31 (23)	
Smoking				.99
No	785 (65)	324 (65)	374 (65)	87 (65)	
Yes	419 (35)	175 (35)	198 (35)	46 (35)	
Alcohol					.44
No	813 (68)	327 (66)	393 (69)	93 (70)	
Yes	391 (32)	172 (34)	179 (31)	40 (30)	
Hypertension					.04
No	1083 (90)	440 (88)	516 (90)	127 (95)	
Yes	121 (10)	59 (12)	56 (10)	6 (5)	
Diabetes					.004
No	1118 (93)	454 (91)	532 (93)	132 (99)	
Yes	86 (7)	45 (9)	40 (7)	1 (1)	
Clonorchis					.87
No	1075 (89)	446 (89)	512 (90)	117 (88)	
Yes	129 (11)	53 (11)	60 (10)	16 (12)	
Cirrhosis					.03
No	631 (52)	282 (57)	278 (49)	71 (53)	
Yes	573 (48)	217 (43)	294 (51)	62 (47)	
Child-Pugh					.27
A	1169 (97)	489 (98)	552 (97)	128 (96)	
B	35 (3)	10 (2)	20 (3)	5 (4)	
Hepatitis background					.008
No	184 (15)	93 (19)	67 (12)	24 (18)	
Hepatitis B	1004 (83)	399 (80)	496 (87)	109 (82)	
Hepatitis C	16 (1)	7 (1)	9 (2)	0 (0)	
Tumor size (cm)					<.001
<5	692 (57)	285 (57)	369 (65)	38 (29)	
≥5	512 (43)	214 (43)	203 (35)	95 (71)	
Tumor number					.003
Single	1076 (89)	462 (93)	493 (86)	121 (91)	
Multiple	128 (11)	37 (7)	79 (14)	12 (9)	
Macroinvasion					<.001
No	1091 (91)	455 (91)	529 (92)	107 (80)	
Yes	113 (9)	44 (9)	43 (8)	26 (20)	
BCLC^a^					<.001
0	124 (10)	45 (9)	76 (13)	3 (2)	
I	875 (73)	377 (76)	405 (71)	93 (70)	
II	90 (7)	32 (6)	47 (8)	11 (8)	
III	115 (10)	45 (9)	44 (8)	26 (20)	
CNLC^b^					<.001
Ia	598 (50)	253 (51)	311 (54)	34 (26)	
Ib	407 (34)	171 (34)	175 (31)	61 (46)	
IIa	55 (5)	18 (4)	30 (5)	7 (5)	
IIb	18 (1)	5 (1)	11 (2)	2 (2)	
IIIa	114 (9)	46 (9)	40 (7)	28 (21)	
IIIb	12 (1)	6 (1)	5 (1)	1 (1)	
AFP^c^ (ng/mL)					<.001
<400	802 (67)	499 (100)	303 (53)	0 (0)	
≥400	402 (33)	0 (0)	269 (47)	133 (100)	
Duration of operation (min)					.06
<240	632 (52)	282 (57)	284 (50)	66 (50)	
≥240	572 (48)	217 (43)	288 (50)	67 (50)	
Bleeding (mL)					.01
<400	618 (51)	267 (54)	299 (52)	52 (39)	
≥400	586 (49)	232 (46)	273 (48)	81 (61)	
Surgical approach					.046
Open	949 (79)	395 (79)	439 (77)	115 (86)	
Minimal	255 (21)	104 (21)	133 (23)	18 (14)	
Radical resection					.03
Yes	799 (66)	332 (67)	392 (69)	75 (56)	
No	405 (34)	167 (33)	180 (31)	58 (44)	
Major resection					<.001
No	977 (81)	421 (84)	471 (82)	85 (64)	
Yes	227 (19)	78 (16)	101 (18)	48 (36)	
MVI^d^					<.001
No	843 (70)	378 (76)	403 (70)	62 (47)	
Yes	361 (30)	121 (24)	169 (30)	71 (53)	
Pathological grade					<.001
Well	76 (6)	57 (11)	19 (3)	0 (0)	
Moderately	1099 (91)	434 (87)	537 (94)	128 (96)	
Poorly	29 (2)	8 (2)	16 (3)	5 (4)	
Follow TACE^e^					<.001
No	1052 (87)	453 (91)	496 (87)	103 (77)	
Yes	152 (13)	46 (9)	76 (13)	30 (23)	

a BCLC: Barcelona Clinic Liver Cancer.

b CNLC: China Clinic Liver Cancer.

c AFP: alpha-fetoprotein.

d MVI: microvascular invasion.

e TACE: transcatheter arterial chemoembolization.

### Ethical Considerations

The data used in this study were obtained from the First Affiliated Hospital of Guangxi Medical University. The procedures followed were in accordance with the ethical standards of the responsible committee on human experimentation (China) and with the WMA Declaration of Helsinki. This study was reviewed and approved by the medical ethics committee of the hospital (2023-E704-01). During postoperative follow-up, informed consent was obtained orally from each participant, and the investigator explained the purpose of the study to the patient or caregiver. The participants were informed of their right to withdraw from the study at any time without penalty or prejudice to their future care, a principle that was strictly upheld throughout the study period. In addition, participants who were interviewed by telephone and completed the postoperative follow-up received a complimentary disease knowledge resource as a token of appreciation and compensation for their participation. All participants’ information was kept confidential, and each patient was assigned an ID to ensure anonymity in data handling and analysis.

## Results

### Risk Stratification

**Figure 1.**
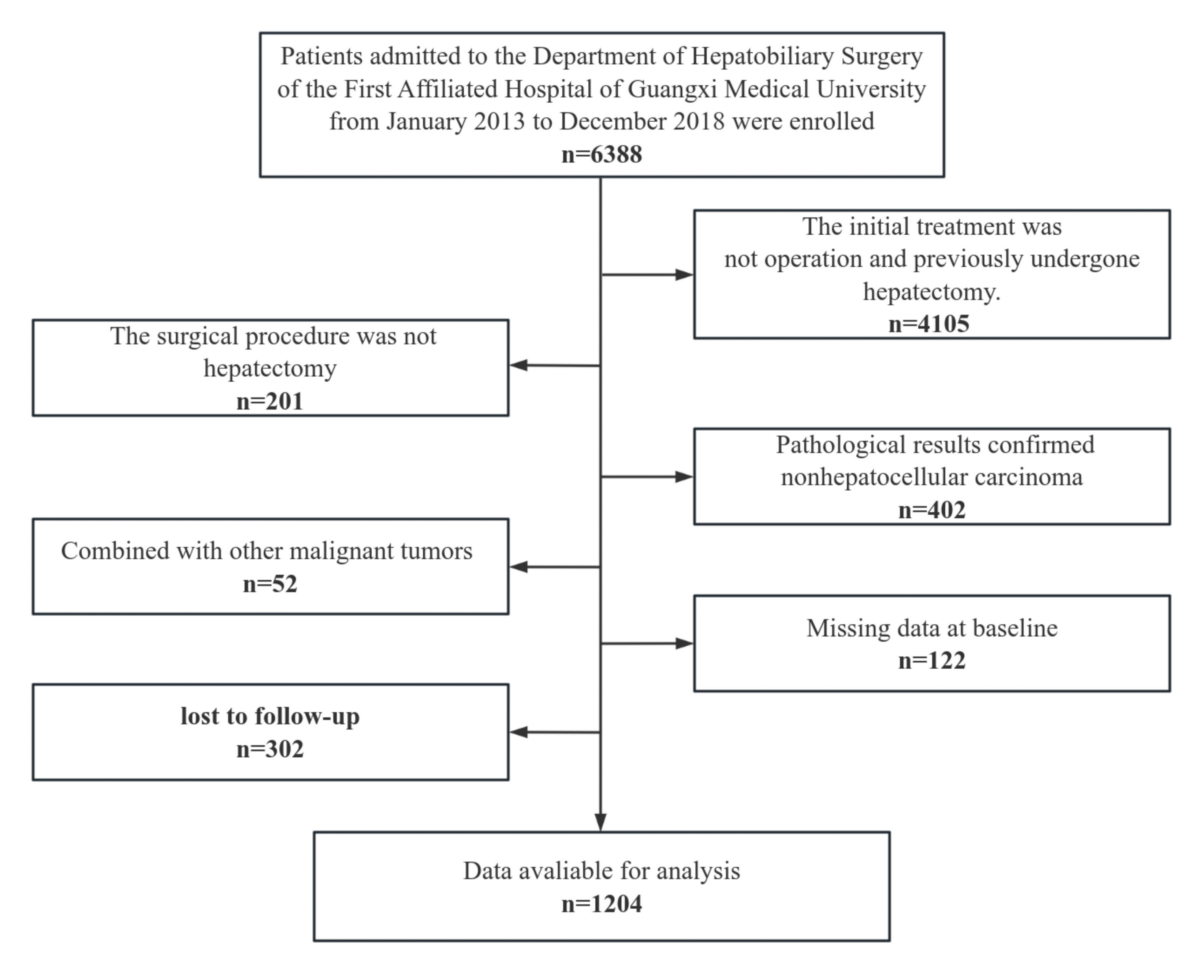
Participant flowchart.

The study included 1204 patients. The participant flowchart is provided in Figure 1. We determined the optimal cutoff ATR level by using X-tile. The patients were divided into low- (OS<873.6; RFS<1469.6) and high-ATR groups (OS>873.6; RFS>1469.6; Figures S1 and S2 in Multimedia Appendix 1). ATR was found to be effective in distinguishing high-risk patients from low-risk patients. We then stratified the ATR cases into low (OS<5.7; RFS<2.8), medium (OS: 5.7‐1469.6; RFS: 2.8‐1469.6), and high (OS>1469.6; RFS>1469.6; Figures S3 and Figure S4 in Multimedia Appendix 1) groups. We found a significant difference in prognosis among the three groups. Stratifying patients into three groups demonstrated higher discriminatory power in predicting prognosis than the two-group classification. The cutoff values of OS and RFS were more closely aligned in the three-group model than in the two-group model. Hence, the patients were categorized into low-, medium-, and high-ATR groups for subsequent analyses.

### Patient Characteristics

**Figure 2.**
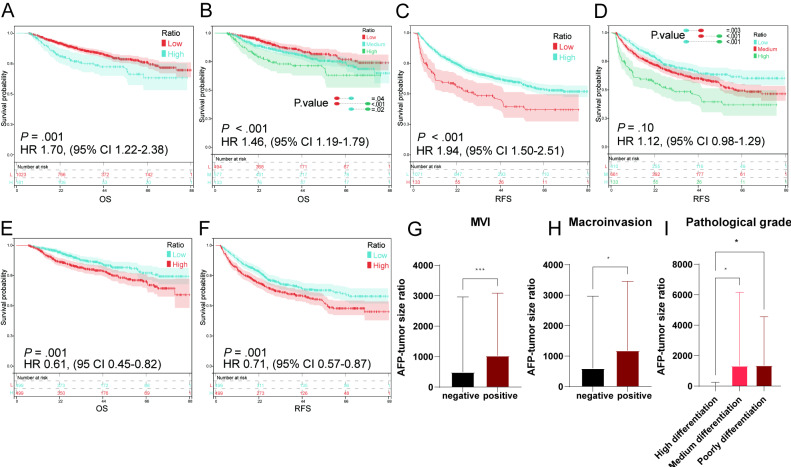
Kaplan-Meier curves estimate the cumulative incidence of HCC recurrence based on ATR and the relationship of ATR and MVI, microinvasion, and tumor differentiation. (A) Survival curves of OS when patients were divided into two groups based on ATR. (B) Survival curves of OS when patients were divided into three groups based on ATR. (C) Survival curves of RFS when patients were divided into two groups based on ATR. (D) Survival curves of RFS when patients were divided into three groups based on ATR. (E) Survival curves of OS when patients were divided into two groups based on ATR after PSM. (F) Survival curves of RFS when patients were divided into two groups based on ATR after PSM. Relationship between (G) ATR and MVI, (H) microinvasion, and (I) tumor differentiation. AFP: alpha-fetoprotein; ATR: alpha-fetoprotein-tumor size ratio; HCC: hepatocellular carcinoma; MVI: microvascular invasion; OS: overall survival; PSM: propensity score matching; RFS: relapse-free survival.

The median OS time was 32.6 (IQR 21.0‐52.1) months, and 444 (36.9%) patients experienced tumor recurrence, and 211 (18.3%) patients died. We found significant differences in OS (Figure 2A,B) and RFS (Figure 2C,D) among patients in the low-, medium-, and high-ATR groups. Moreover, ATR was negatively correlated with patient prognosis.

Table 1 summarizes the clinical characteristics and surgical variables of patients stratified according to serum ATR level. Differences in gender, BMI, diabetes mellitus, background of liver disease, tumor size, tumor number, MVI, BCLC staging, CNLC staging, AFP level, and surgical bleeding were statistically significant among the three groups (*P<.*05).

The proportion of female patients, macrovascular invasion, MVI, AFP>400 ng/mL, and advanced stages according to the BCLC and CNLC staging systems increased in the high-ATR group. However, the proportion of patients with radical resection, well-differentiated tumor, and BMI>24 was lower in this group.

### ATR is an Independent Risk Factor for OS and RFS in Patients With HCC Who Underwent Hepatectomy

We performed univariate Cox analyses on clinicopathologic parameters. Gender, cirrhosis, Child-Pugh classification, tumor size, tumor number, macrovascular invasion, BCLC staging, CNLC staging, surgical approach, bleeding, radical resection, MVI, postoperative TACE, and ATR were statistically significant contributors to OS and RFS. Variables significantly associated with prognosis in univariate Cox regression analysis were included in multivariate Cox regression analysis.

For OS, cirrhosis (HR 1.47, 95% CI 1.11‐1.96; *P*=.008), CNLC stages IIIa (HR 4.52, 95% CI 1.48‐13.82; *P*=.008) and IIIb (HR 5.04, 95% CI 1.43‐17.76; *P*=.01), bleeding volume (HR 1.47, 95% CI 1.10‐1.97; *P*=.009), postoperative TACE (HR 4.38, 95% CI 3.22‐5.97; *P*<.001), medium-ATR group (HR 1.41, 95% CI 1.03‐1.94; *P*=.03), and high-ATR group (HR 1.59, 95% CI 1.02‐2.47; *P*=.04) showed statistically significant results (Table 2).

**Table 2.** Univariate and multivariate Cox regression analyses of the associations between the prognostic factors and the overall survival of the patients with HCC^a^.

Variables		Stats, n (%)	HR^b^ (95% CI; univariable)	*P* value	HR (95% CI; multivariable)	*P* value
Sex						
Female		187 (15.5)	1 (reference)	—^c^	1 (reference)	—
Male		1017 (84.5)	1.56 (1.01‐2.41)	.04	1.36 (0.87‐2.11)	.17
Age (years)						
<60		963 (80)	1 (reference)	—	—	—
≥60		241 (20)	0.76 (0.53‐1.09)	.14	—	—
BMI						
<24		791 (65.7)	1 (reference)	—	—	—
≥24		413 (34.3)	1.05 (0.79‐1.39)	.75	—	—
Cirrhosis						
No		631 (52.4)	1 (reference)	—	1 (reference)	—
Yes		573 (47.6)	1.49 (1.14‐1.96)	.004	1.47 (1.11‐1.96)	.008
Child-Pugh						
A		1169 (97.1)	1 (reference)	—	1 (reference)	—
B		35 (2.9)	1.98 (1.08‐3.64)	.03	1.25 (0.66‐2.35)	.495
Hepatitis background						
No		184 (15.3)	1 (reference)	—	—	—
B		1004 (83.4)	1.27 (0.86‐1.90)	.23	—	—
C		16 (1.3)	0.77 (0.18‐3.21)	.71	—	—
Tumor size (cm)						
<5		692 (57.5)	1 (reference)	—	1	—
≥5		512 (42.5)	1.74 (1.33‐2.29)	<.001	1.04 (0.66‐1.64)	.87
Tumor number						
Single		1076 (89.4)	1 (reference)	—	1 (reference)	—
Multiple		128 (10.6)	1.93 (1.35‐2.77)	<.001	0.72 (0.38‐1.35)	.31
Macroinvasion						
No		1091 (90.6)	1 (reference)	—	1 (reference)	—
Yes		113 (9.4)	2.57 (1.71‐3.87)	<.001	0.27 (0.03‐2.70)	.27
BCLC^d^						
0		124 (10.3)	1 (reference)	—	1 (reference)	—
I		875 (72.7)	2.38 (1.26‐4.52)	.008	1.88 (0.96‐3.68)	.06
II		90 (7.5)	4.27 (2.04‐8.93)	<.001	1.31 (0.36‐4.67)	.68
III		115 (9.6)	6.21 (3.00‐12.83)	<.001	3.97 (0.48‐32.91)	.20
CNLC^e^						
Ia		598 (49.7)	1 (reference)	—	1 (reference)	—
Ib		407 (33.8)	1.68 (1.23‐2.30)	.001	1.35 (0.81‐2.25)	.24
IIa		55 (4.6)	2.31 (1.30‐4.08)	.004	1.77 (0.51‐6.10)	.37
IIb		18 (1.5)	3.13 (1.36‐7.18)	.007	3.23 (0.81‐12.91)	.09
IIIa		114 (9.5)	3.84 (2.49‐5.92)	<.001	4.52 (1.48‐13.82)	.008
IIIb		12 (1)	4.70 (2.04‐10.83)	<.001	5.04 (1.43‐17.76)	.01
Surgical approach						
Open		949 (78.8)	1 (reference)	—	1 (reference)	—
Minimal		255 (21.2)	0.64 (0.42‐0.97)	.03	0.99 (0.64‐1.52)	.95
Bleeding (mL)						
<400		618 (51.3)	1 (reference)	—	1 (reference)	—
≥400		586 (48.7)	1.88 (1.42‐2.49)	<.001	1.47 (1.10‐1.97**)**	.009
Radical resection						
Yes		799 (66.4)	1 (reference)	—	1 (reference)	—
No		405 (33.6)	1.65 (1.26‐2.17)	<.001	1.21 (0.91‐1.61)	.19
MVI^f^						
No		843 (70)	1 (reference)	—	1 (reference)	—
Yes		361 (30)	1.62 (1.23‐2.14)	<.001	1.06 (0.77‐1.44)	.73
Pathological grade						
Well		76 (6.3)	1 (reference)	—	—	—
Moderately		1099 (91.3)	1.41 (0.77‐2.59)	.27	—	—
Poorly		29 (2.4)	0.86 (0.27‐2.69)	.79	—	—
AFP^g^ (ng/mL)						
<400		802 (66.6)	1 (reference)	—	—	—
≥400		402 (33.4)	1.18 (0.89‐1.56)	.25	—	—
Follow TACE^h^						
No		1052 (87.4)	1 (reference)	—	1 (reference)	—
Yes		152 (12.6)	5.22 (3.94‐6.92)	<.001	4.38 (3.22‐5.97)	<.001
AFP-size ratio						
Low		499 (41.4)	1 (reference)	—	1 (reference)	—
Medium		572 (47.5)	1.41 (1.04‐1.91)	.03	1.41 (1.03‐1.94)	.03
High		133 (11)	2.22 (1.46‐3.36)	<.001	1.59 (1.02‐2.47)	.04

a HCC: hepatocellular carcinoma.

b HR: hazard ratio.

c Not applicable.

d BCLC: Barcelona Clinic Liver Cancer.

e CNLC: China Clinic Liver Cancer.

f MVI: microvascular invasion.

g AFP: alpha-fetoprotein.

h TACE: transcatheter arterial chemoembolization.

For RFS, gender (HR 1.48, 95% CI 1.09‐2.02; *P*=.01), cirrhosis (HR 1.26, 95% CI 1.04‐1.54; *P*=.02), MVI (HR 1.27, 95% CI 1.03‐1.57; *P*=.03), postoperative TACE (HR 8.20, 95% CI 6.50‐10.33; *P*<.001), medium-ATR group (HR 1.33, 95% CI 1.03‐1.70; *P*=.03), and high-ATR group (HR 2.10, 95% CI 1.40‐3.15; *P*<.001) showed statistically significant results (Table 3). ATR was statistically significant in OS and RFS. Hence, ATR was regarded as an independent risk factor for the prognosis of patients with HCC who underwent hepatectomy.

**Table 3.** Univariate and multivariate Cox regression analyses of the associations between the prognostic factors and the relapse-free survival of the patients with HCC^a^.

Variables		Stats, n (%)	HR^b^ (95% CI; univariable)	*P* value	HR (95% CI; multivariable)	*P* value
Sex						
Female		187 (15.5)	1 (reference)	—^c^	1 (reference)	—
Male		1017 (84.5)	1.72 (1.27‐2.33)	<.001	1.48 (1.09‐2.02)	.01
Age (years)						
<60		963 (80)	1 (reference)	—	—	—
≥60		241 (20)	0.92 (0.72‐1.16)	46	—	—
BMI						
<24		791 (65.7)	1 (reference)	—	—	—
≥24		413 (34.3)	1.03 (0.85‐1.25)	.78	—	—
Cirrhosis						
No		631 (52.4)	—	—	1 (reference)	—
Yes		573 (47.6)	1.36 (1.13‐1.64)	.001	1.26 (1.04‐1.54)	.02
Child-Pugh						
A		1169 (97.1)	1 (reference)	—	1 (reference)	—
B		35 (2.9)	1.97 (1.27‐3.06)	.002	1.25 (0.80‐1.97)	.33
Hepatitis background						
No		184 (15.3)	1 (reference)	—	—	—
Hepatitis B		1004 (83.4)	1.31 (0.99‐1.73)	.05	—	—
Hepatitis C		16 (1.3)	1.33 (0.57‐3.08)	.51	—	—
Tumor size (cm)						
<5		692 (57.5)	1 (reference)	—	1 (reference)	—
≥5		512 (42.5)	1.48 (1.23‐1.78)	<.001	1.06 (0.77‐1.48)	.71
Tumor number						
Single		1076 (89.4)	1 (reference)	—	1 (reference)	—
Multiple		128 (10.6)	2.61 (2.05‐3.32)	<.001	1.44 (0.92‐2.24)	.11
Macroinvasion						
No		1091 (90.6)	1 (reference)	—	1 (reference)	—
Yes		113 (9.4)	1.43 (1.03‐2.00)	.03	0.31 (0.05‐2.08)	.23
BCLC^d^						
0		124 (10.3)	1 (reference)	—	1 (reference)	—
I		875 (72.7)	1.39 (0.98‐1.96)	.06	1.12 (0.77‐1.64)	.54
II		90 (7.5)	3.36 (2.21‐5.09)	<.001	1.48 (0.73‐3.00)	.28
III		115 (9.6)	2.21 (1.41‐3.47)	<.001	2.28 (0.52‐10.04)	.28
CNLC^e^						
Ia		598 (49.7)	1 (reference)	—	1 (reference)	—
Ib		407 (33.8)	1.66 (1.34‐2.05)	<.001	1.24 (0.86‐1.79)	.24
IIa		55 (4.6)	3.08 (2.15‐4.41)	<.001	0.71 (0.31‐1.65)	.43
IIb		18 (1.5)	3.67 (1.99‐6.75)	<.001	1.35 (0.52‐3.53)	.54
IIIa		114 (9.5)	2.12 (1.51‐2.99)	<.001	2.23 (0.67‐7.42)	.19
IIIb		12 (1.0)	2.45 (1.15‐5.22)	.02	0.74 (0.26‐2.14)	.58
Surgical approach						
Open		949 (78.8)	1 (reference)	—	1	—
Minimal		255 (21.2)	0.75 (0.58‐0.97)	.03	1.12 (0.86‐1.47)	.41
Bleeding (mL)						
<400		618 (51.3)	1 (reference)	—	1 (reference)	—
≥400		586 (48.7)	1.63 (1.35‐1.96)	<.001	1.16 (0.95‐1.42)	.14
Radical resection						
Yes		799 (66.4)	1 (reference)	—	1 (reference)	—
No		405 (33.6)	1.46 (1.20‐1.76)	<.001	1.18 (0.96‐1.44)	.11
MVI^f^						
No		843 (70)	1 (reference)	—	1 (reference)	—
Yes		361 (30)	1.59 (1.31‐1.93)	<.001	1.27 (1.03‐1.57)	.03
Pathological grade						
Well		76 (6.3)	1 (reference)	—	—	—
Moderately		1099 (91.3)	1.45 (0.95‐2.20)	09	—	—
Poorly		29 (2.4)	0.96 (0.44‐2.08)	.92	—	—
AFP^g^ (ng/mL)						
<400		802 (66.6)	1 (reference)	—	1 (reference)	—
≥400		402 (33.4)	1.27 (1.05‐1.54)	.01	0.97 (0.75‐1.26)	.82
Follow-up TACE^h^						
No		1052 (87.4)	1 (reference)	—	1 (reference)	—
Yes		152 (12.6)	10.05 (8.15‐12.38)	<.001	8.20 (6.50‐10.33)	<.001
AFP-size ratio						
Low		410 (34.1)	1 (reference)	—	1	—
Medium		661 (54.9)	1.35 (1.09‐1.67)	006	1.33 (1.03‐1.70)	.03
High		133 (11)	2.35 (1.75‐3.16)	<.001	2.10 (1.40‐3.15)	<.001

a HCC: hepatocellular carcinoma.

b HR: hazard ratio.

c Not applicable.

d BCLC: Barcelona Clinic Liver Cancer.

e CNLC: China Clinic Liver Cancer.

f MVI: microvascular invasion.

g AFP: alpha-fetoprotein.

h TACE: transcatheter arterial chemoembolization.

Figure S5A,B in Multimedia Appendix 1 displays the distribution trend of ATR in the OS and RFS of patients with HCC, indicating the potential of ATR to categorize patients into three distinct groups. Figure S5C,D in Multimedia Appendix 1 illustrates the distribution of ATR and its correlation trend with prognosis in the OS and RFS of patients with HCC, revealing a negative correlation between ATR levels and patient prognosis.

### PSM Cohort Analysis

PSM was performed to validate the findings and adjust the effect of confounding factors. We performed 1:1 PSM on patients in the low-ATR group. After PSM, most variables were controlled, and significant differences in MVI and degree of pathological grade remained (Table S1 in Multimedia Appendix 1). Univariate and multivariate Cox analyses were also conducted. The results revealed that ATR was statistically significant in OS (HR 1.30, 95% CI 1.05‐1.61; *P*=.02; Figure 2E and Table S2 in Multimedia Appendix 1) and RFS (HR 1.61, 95% CI 1.18‐2.19; *P*=.008; Figure 2E,F and Table S3 in Multimedia Appendix 1), demonstrating that ATR is an independent risk factor for the prognosis of patients with HCC who underwent hepatectomy following PSM. These results corroborate the conclusion. Although we performed PSM, significant differences in MVI and tumor differentiation were still found.

### Correlation of ATR With Tumor Malignancy Level

MVI and poor pathological grade are recognized as independent risk factors for an unfavorable prognosis in patients with HCC. We analyzed the relationship of MVI, pathological grade, and macrovascular invasion with ATR. An elevated ATR was significantly associated with positive MVI, macrovascular invasion, and poor tumor differentiation (Figure 2G-I). These findings support the notion that in HCC cases with comparable tumor sizes, a poor prognosis is associated with elevated levels of AFP.

### Construction of a Prognostic Model for HCC Based on ATR

**Figure 3.**
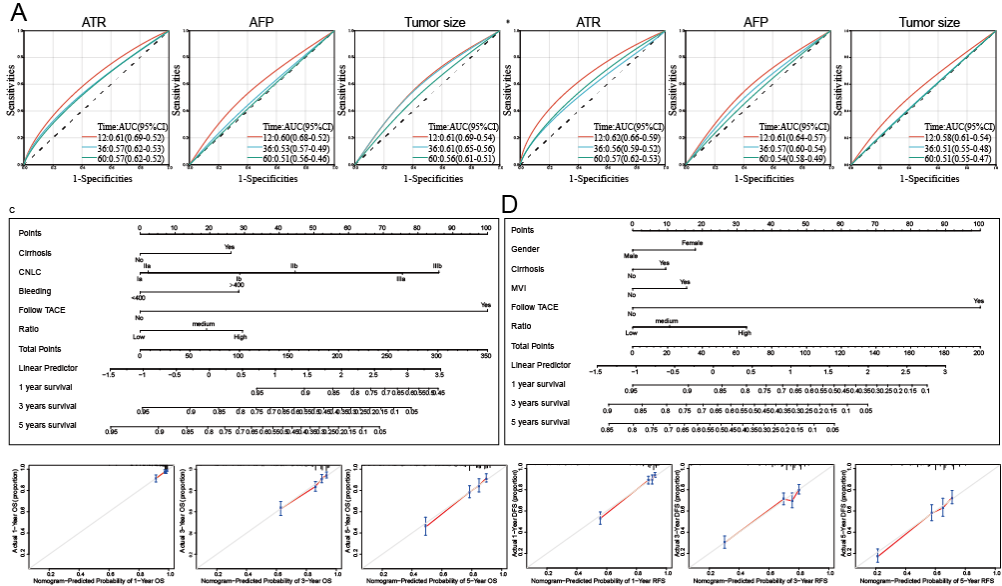
Prediction model of patients with HCC following surgical treatment. (A) The ROC curve of ATR, AFP, and tumor size was used to predict the OS of patients. (B) The ROC curve of ATR, AFP, and tumor size was used to predict the RFS of patients. (C) Nomogram and calibration curve for the OS of patients. (D) Nomogram and calibration curve for the RFS of patients. AFP: alpha-fetoprotein; ATR: alpha-fetoprotein-tumor size ratio; CNLC: China Clinic Liver Cancer; HCC: hepatocellular carcinoma; MVI: microvascular invasion; OS: overall survival; RFS: relapse-free survival; ROC: receiver operating characteristic; TACE: transcatheter arterial chemoembolization.

To enhance the foundation for clinical decision-making, we developed a prediction model. We found that ATR was more effective in predicting OS and RFS than AFP or tumor size alone (Figure 3A,B). We developed a prognostic model using the ATR. In the prediction model for OS, the C-index of the model was 0.73, achieving moderate predictive efficacy. The correction curve confirmed that the model predictions were stable (Figure 3C). In the prediction model for RFS, the C-index of the model was 0.73, achieving moderate predictive efficacy, and the correction curves confirmed that the model predictions were stable (Figure 3D).

## Discussion

### Principal Findings

This study demonstrated that the ATR is an independent risk factor for OS and RFS in patients with HCC who underwent hepatectomy. The prognostic model based on the ATR was found to be effective in predicting prognosis. Moreover, ATR was positively correlated with the degree of HCC malignancy.

The cutoff values of OS and RFS in the low- and medium-ATR groups were 5.7 and 2.8, respectively, and the OS and RFS in the medium- and high-ATR groups shared the same cutoff value of 1469.6. The cutoff values for OS and RFS were identical in the medium- and high-ATR groups, and the difference between the groups was minimal. This result indicates that the ATR can effectively and stably predict patient prognosis. According to the HCC data statistics, the recurrence rate of patients with HCC following surgery is higher than the mortality rate, and the causes of recurrence and mortality are highly heterogeneous. Thus, differences in cutoff values are often observed in the prediction of postoperative OS and RFS of patients with HCC [3]. Our results demonstrated small differences in the cutoff values of OS and RFS between the low- and medium-ATR groups.

### Comparison to Prior Work

Elevated AFP and increased tumor size are prognostic risk factors for HCC, and the combination of tumor burden and AFP serves as an important indicator of poor prognosis [14,15]. However, the potential correlation between AFP and the number of tumor cells is often overlooked. We found that AFP and tumor size showed a statistically significant association in univariate Cox analysis. However, in Cox multivariate regression analysis, AFP and tumor size exhibited no statistically significant association with prognosis. Hence, compared with AFP or tumor size, ATR may be a more suitable prognostic marker.

Notably, AFP levels have been associated with MVI [16], macrovascular invasion, and poor tumor differentiation [17,18]. In this study, MVI, macrovascular invasion, and poor tumor differentiation were found to be associated with elevated ATR levels. This result shows that the ATR is an effective marker for assessing the degree of malignancy in HCC. Therefore, this study offers a theoretical basis for identifying the malignancy of small HCC tumors and supports informed medical decision-making.

Many in vitro studies have shown that AFP is associated with tumor progression and metastasis. AFP can inhibit programmed death by inhibiting the Fas/FADD apoptotic pathway and activating the PI3K/AKT signaling pathway [12,19]. Moreover, it plays an important role in the metastasis of HCC [20] and can induce tumor immunosuppression and evade immune surveillance [21,22]. AFP levels are elevated in response to the biological behavior of HCC, contributing to tumor proliferation, invasion, and metastasis. However, the relationship between the amount of AFP secreted by HCC cells per unit volume and the degree of tumor malignancy and patient prognosis remains unclear. This study demonstrates that an increase in AFP secreted by HCC cells per unit volume is associated with a high degree of malignancy in HCC and poor patient prognosis.

We used the ATR to construct a nomogram, and the model effectively predicted the prognosis of patients with HCC. This study introduced a novel approach involving the use of the ATR for prognostic prediction in patients with HCC following hepatectomy. Previous studies have proposed a pre- to postoperative AFP ratio-based nomograms for the prognostic assessment of patients with HCC, yielding an area under curve of approximately 0.72 [23]. However, these prognostic models demonstrated limited efficacy in patients with AFP-negative.

Cirrhosis is a risk factor for poor postoperative prognosis in patients with HCC [24,25]. The association of intraoperative bleeding with poor patient prognosis has been well established; moreover, intraoperative bleeding has been associated with perioperative blood transfusion, which has been shown to be associated with poor patient prognosis [26,27]. Gender factors are also associated with the prognosis of patients with HCC, with men having a higher risk of recurrence than women [4]. We included cirrhosis and bleeding in the model for predicting OS, in addition to gender, cirrhosis, and MVI, in the model for predicting RFS. Both models showed good predictive efficacy.

### Strengths

This study uses the ATR to predict the prognosis of patients with HCC, and a prognostic prediction model was constructed using the ATR, which provides a theoretical basis for clinical prognosis prediction, recurrence risk stratification, and postoperative follow-up strategies.

### Limitations

First, most of the patients included in this study were patients with hepatitis B virus–related HCC. Second, uncontrollable bias possibly existed despite the matching of MVI and pathological grade in PSM.

### Conclusions

ATR was demonstrated to be an independent risk factor for prognosis in patients with HCC undergoing surgery and is associated with MVI, macrovascular invasion, and poor tumor differentiation. Hence, the ATR can be considered a promising prognostic marker for HCC.

## Supplementary material

10.2196/64929 Multimedia Appendix 1 Supplementary figures.

## Abbreviations

AFP: alpha-fetoprotein
ATR: alpha-fetoprotein-tumor size ratio
BCLC: Barcelona Clinic Liver Cancer
CNLC: China Clinic Liver Cancer
HCC: hepatocellular carcinoma
HR: hazard ratio
MVI: microvascular invasion
OS: overall survival
PSM: propensity score matching
RFS: relapse-free survival
TACE: transcatheter arterial chemoembolization
