# Supplementary material for: Serum Alpha-Fetoprotein-Tumor Size Ratio as a Prognostic Marker After Hepatic Resection for Primary Hepatocellular Carcinoma: Propensity Score Matched Retrospective Cohort Study
Source: JMIR Cancer. 2025 Aug 26;11:e64929. doi: 10.2196/64929 (PMC12380365; doi:10.2196/64929)
Supplement: Multimedia Appendix 1 [file cancer-v11-e64929-s001.pdf]

Figure S1

Survival Analysis: ratio

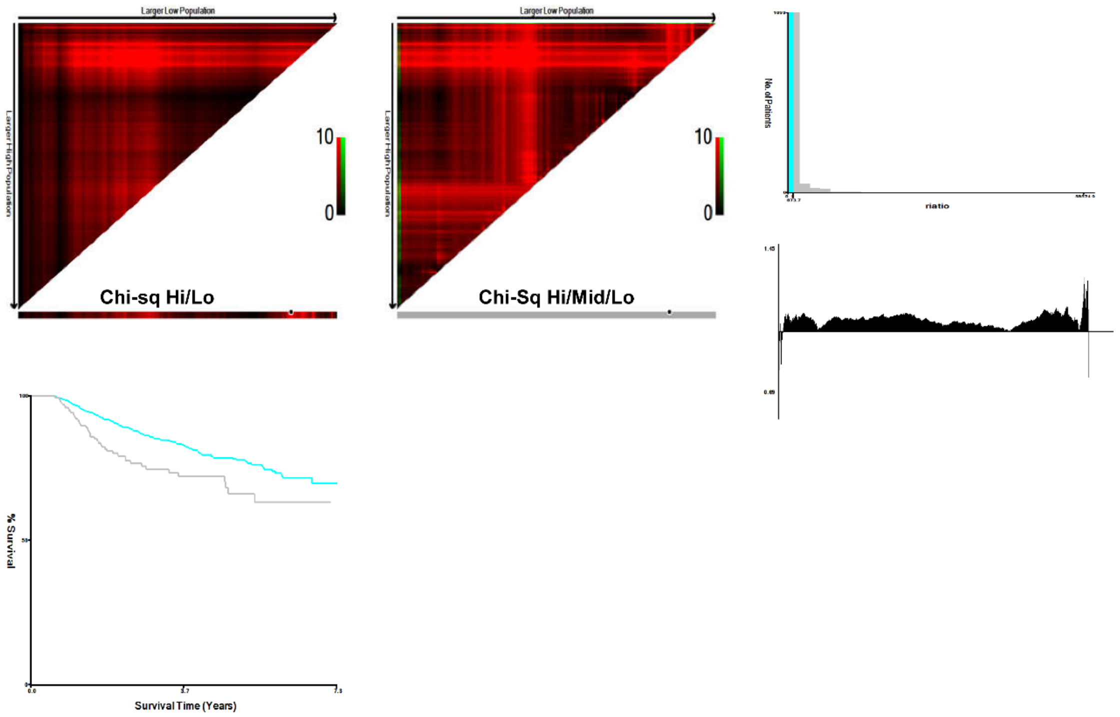

Subpopulation Cutpoints:

Pt No	% Total	Events	Rate	Rank	Range
1026	85.22	168	16.37	0 to 1011	0.08 thru 873.65
178	14.78	43	24.16	1012 to 1181	905.68 thru 59574.00
1204	100.00	211	17.52	0 to 1181	0.08 thru 59574.00

Statistics:

Variable	Value	
Miller-Seigmund P	0.0309	Max: 0.0309
Chi-sq Hi/Lo	10.3589	Max: 10.3589
Relative Risk 1 vs 2	1.00 / 1.48	

Figure S2

Survival Analysis: ratio

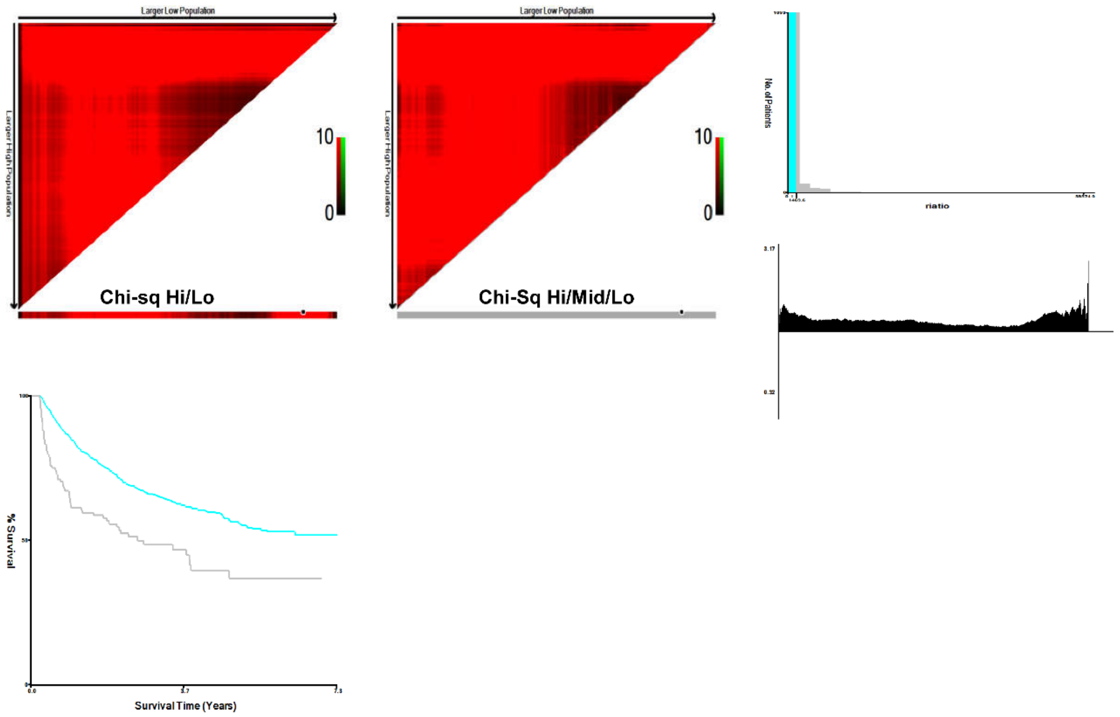

Subpopulation Cutpoints:

Pt No	% Total	Events	Rate	Rank	Range
1071	88.95	376	35.11	0 to 1056	0.08 thru 1469.62
133	11.05	68	51.13	1057 to 1181	1469.67 thru 59574.00
1204	100.00	444	36.88	0 to 1181	0.08 thru 59574.00

Statistics:

Variable	Value	
Miller-Seigmund P	<0.0001	Max: <0.0001
Chi-sq Hi/Lo	26.2290	Max: 26.2290
Relative Risk 1 vs 2	1.00 / 1.46	

Figure S3

Survival Analysis: riatio

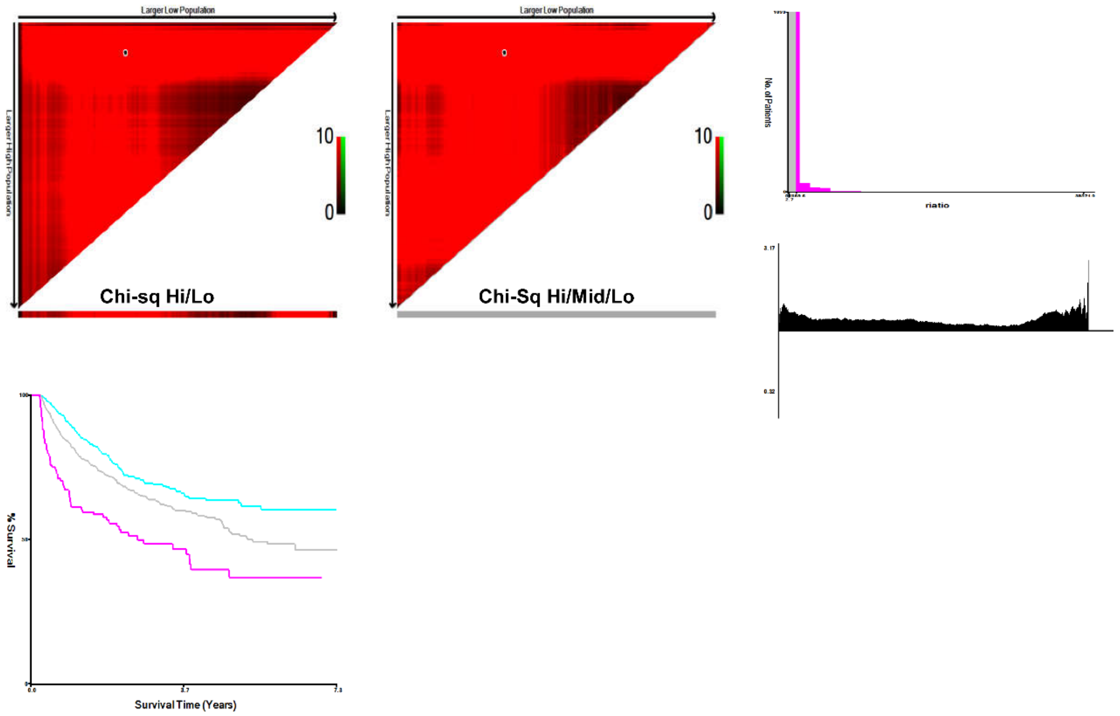

Subpopulation Cutpoints:

Pt No	% Total	Events	Rate	Rank	Range
411	34.14	123	29.93	0 to 398	0.08 thru 2.75
660	54.82	253	38.33	399 to 1056	2.77 thru 1469.62
133	11.05	68	51.13	1057 to 1181	1469.67 thru 59574.00
1204	100.00	444	36.88	0 to 1181	0.08 thru 59574.00

Statistics:

Variable	Value	
Chi-Sq Hi/Mid/Lo	33.3312	Max: 33.9956
Lo vs Mid	7.8060	
Mid vs Hi	16.2087	
Lo vs Hi	33.4590	
Relative Risk 1 vs 2 vs 3	1.00 / 1.28 / 1.71	

Figure S4

Survival Analysis: riatio

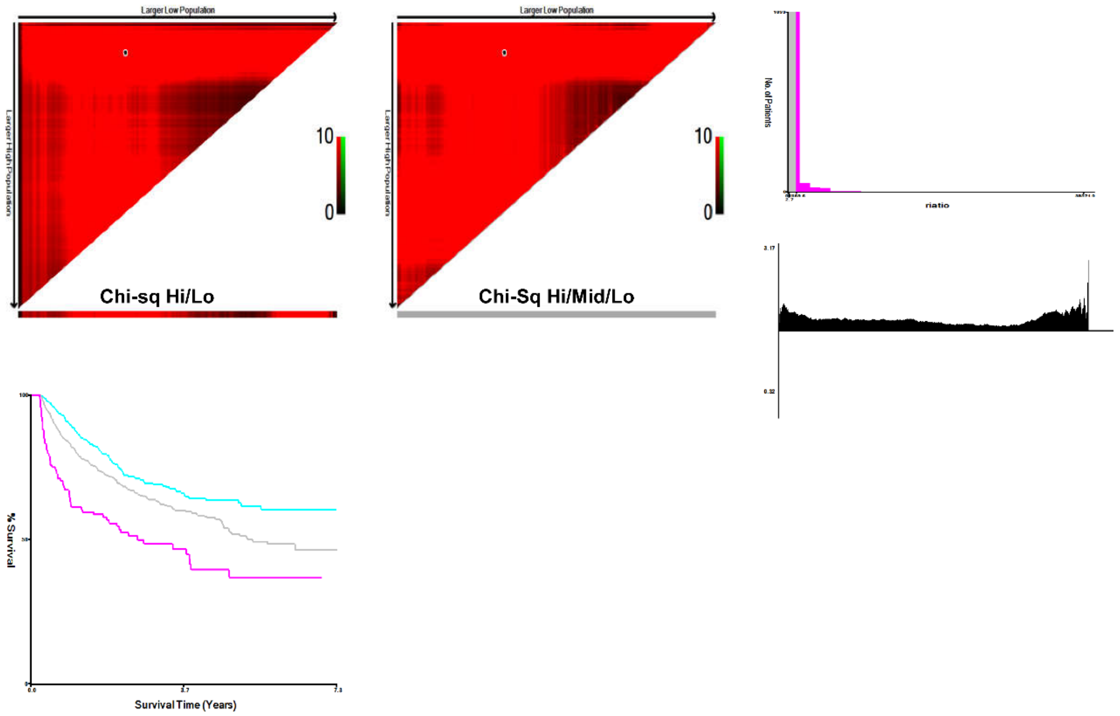

Subpopulation Cutpoints:

Pt No	% Total	Events	Rate	Rank	Range
411	34.14	123	29.93	0 to 398	0.08 thru 2.75
660	54.82	253	38.33	399 to 1056	2.77 thru 1469.62
133	11.05	68	51.13	1057 to 1181	1469.67 thru 59574.00
1204	100.00	444	36.88	0 to 1181	0.08 thru 59574.00

Statistics:

Variable	Value	
Chi-Sq Hi/Mid/Lo	33.3312	Max: 33.9956
Lo vs Mid	7.8060	
Mid vs Hi	16.2087	
Lo vs Hi	33.4590	
Relative Risk 1 vs 2 vs 3	1.00 / 1.28 / 1.71	

Figure S5

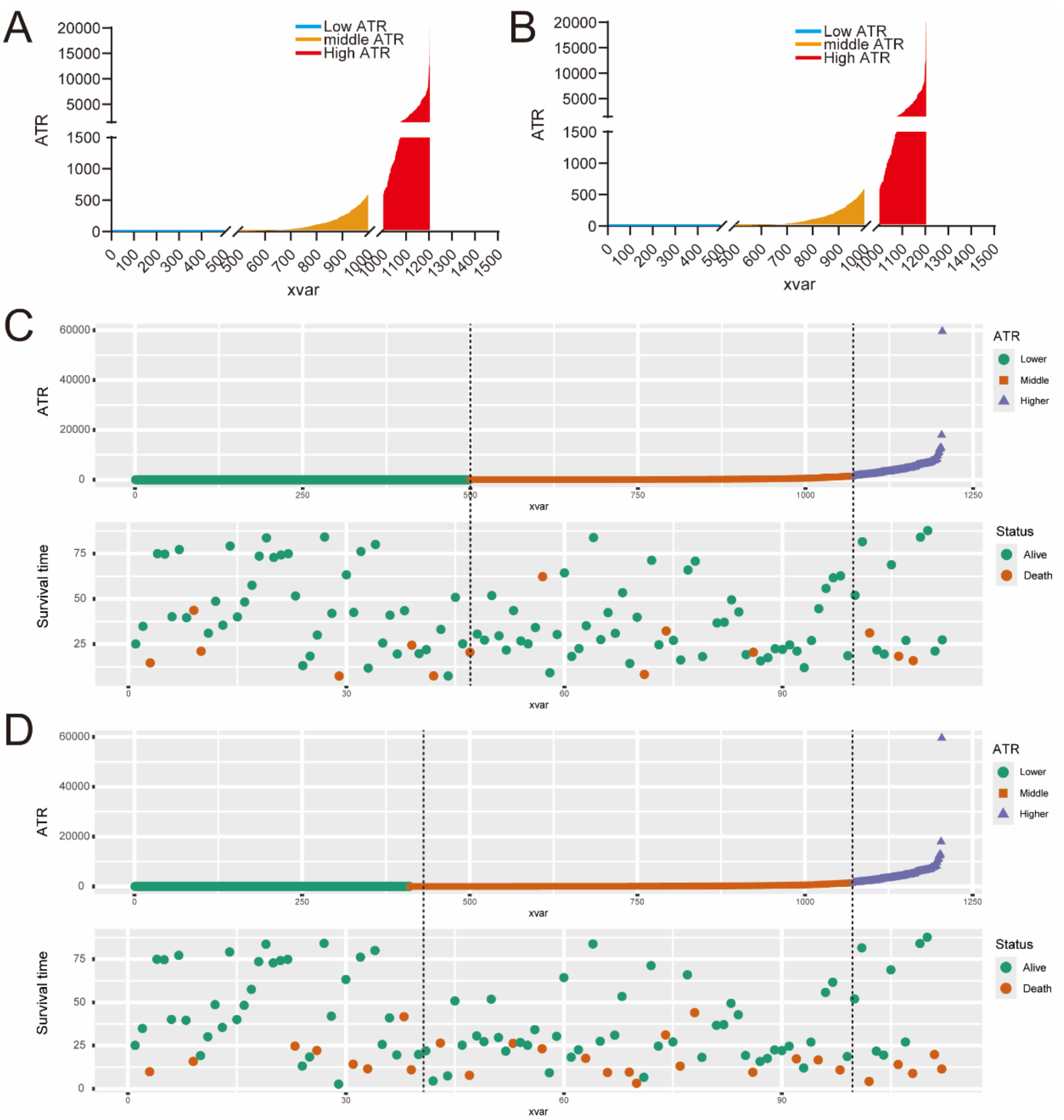
A
B
C
D
Low ATR
middle ATR
High ATR
ATR
xvar
Survival time
Lower
Middle
Higher
Status
Alive
Death

Table S1 The patient's baseline data after PSM

Variables	Total (n = 998)	Low (n = 499)	High (n = 499)	P-value
Gender, n (%)				0.563
Female	123 (12)	58 (12)	65 (13)	
Male	875 (88)	441 (88)	434 (87)	
Age(year), n (%)				0.309
<60	792 (79)	389 (78)	403 (81)	
≥60	206 (21)	110 (22)	96 (19)	
BMI, n (%)				0.057
<24	630 (63)	300 (60)	330 (66)	
≥24	368 (37)	199 (40)	169 (34)	
Smoking, n (%)				0.791
No	643 (64)	324 (65)	319 (64)	
Yes	355 (36)	175 (35)	180 (36)	
Alcohol, n (%)				0.592
No	663 (66)	327 (66)	336 (67)	
Yes	335 (34)	172 (34)	163 (33)	
Hypertension, n (%)				0.216
No	893 (89)	440 (88)	453 (91)	
Yes	105 (11)	59 (12)	46 (9)	
Diabetes, n (%)				0.42
No	916 (92)	454 (91)	462 (93)	
Yes	82 (8)	45 (9)	37 (7)	
Clonorchis, n (%)				0.839
No	889 (89)	446 (89)	443 (89)	
Yes	109 (11)	53 (11)	56 (11)	
Cirrhosis, n (%)				0.566
No	554 (56)	282 (57)	272 (55)	
Yes	444 (44)	217 (43)	227 (45)	
Child-Pugh, n (%)				0.132
A	969 (97)	489 (98)	480 (96)	
B	29 (3)	10 (2)	19 (4)	
Hepatitis background, n (%)				0.025
No	155 (16)	93 (19)	62 (12)	
Hepatitis B	828 (83)	399 (80)	429 (86)	
Hepatitis C	15 (2)	7 (1)	8 (2)	
Tumor size(cm), n (%)				0.112
<5	544 (55)	285 (57)	259 (52)	
≥5	454 (45)	214 (43)	240 (48)	
Tumor number, n (%)				0.305
Single	914 (92)	462 (93)	452 (91)	
Multiple	84 (8)	37 (7)	47 (9)	
Macroinvasion, n (%)				0.145
No	895 (90)	455 (91)	440 (88)	
Yes	103 (10)	44 (9)	59 (12)	
BCLC, n (%)				0.349
0	97 (10)	45 (9)	52 (10)	
I	735 (74)	377 (76)	358 (72)	
II	61 (6)	32 (6)	29 (6)	
III	105 (11)	45 (9)	60 (12)	

CNLC, n (%)				0.377
Ia	473 (47)	253 (51)	220 (44)	
Ib	364 (36)	171 (34)	193 (39)	
IIa	37 (4)	18 (4)	19 (4)	
IIb	9 (1)	5 (1)	4 (1)	
IIIa	104 (10)	46 (9)	58 (12)	
IIIb	11 (1)	6 (1)	5 (1)	
Duration of operation(min), n (%)				0.076
<240	535 (54)	282 (57)	253 (51)	
≥240	463 (46)	217 (43)	246 (49)	
Bleeding(ml), n (%)				0.254
<400	515 (52)	267 (54)	248 (50)	
≥400	483 (48)	232 (46)	251 (50)	
Surgical approach, n (%)				1
Open	789 (79)	395 (79)	394 (79)	
Minimal	209 (21)	104 (21)	105 (21)	
Radical resection, n (%)				0.32
Yes	648 (65)	332 (67)	316 (63)	
No	350 (35)	167 (33)	183 (37)	
Major resection, n (%)				0.069
No	819 (82)	421 (84)	398 (80)	
Yes	179 (18)	78 (16)	101 (20)	
MVI, n (%)				**0.011**
No	719 (72)	378 (76)	341 (68)	
Yes	279 (28)	121 (24)	158 (32)	
Pathological grade, n (%)				**< 0.001**
Well	76 (8)	57 (11)	19 (4)	
Moderately	908 (91)	434 (87)	474 (95)	
Poorly	14 (1)	8 (2)	6 (1)	
Follow TACE, n (%)				0.152
No	891 (89)	453 (91)	438 (88)	
Yes	107 (11)	46 (9)	61 (12)	

Abbreviations: BMI, Body mass index; BCLC, Barcelona Clinic Liver Cancer; CNLC, China Clinic Liver Cancer; AFP, alpha-fetoprotein; MVI, microvascular invasion, TACE, transcatheter arterial chemoembolization.

Table S2 Univariate and multivariate Cox regression analyses of the associations between the prognostic factors and the relapse-free survival of the HCC patients after PSM

Variables	desc	stats	HR (univariable)	HR (multivariable)
Gender	Male	123 (12.3%)		
	Female	875 (87.7%)	2.00 (1.33-3.00, p<.001)	1.47 (0.97-2.22, p=.067)
Age (years)	<60	792 (79.4%)		
	≥60	206 (20.6%)	0.96 (0.74-1.25, p=.773)	
BMI	<24	630 (63.1%)		
	≥24	368 (36.9%)	0.99 (0.80-1.23, p=.949)	
Cirrhosis	No	554 (55.5%)		
	Yes	444 (44.5%)	1.33 (1.08-1.64, p=.007)	**1.26 (1.01-1.57, p=.038)**
Child-Pugh	A	969 (97.1%)		
	B	29 (2.9%)	1.75 (1.06-2.89, p=.029)	1.24 (0.75-2.07, p=.405)
Hepatitis background	No	155 (15.5%)		
	Hepatitis B	828 (83.0%)	1.20 (0.89-1.63, p=.230)	
	Hepatitis C	15 (1.5%)	1.35 (0.58-3.14, p=.492)	
Tumor size(cm)	<5	544 (54.5%)		
	≥5	454 (45.5%)	1.42 (1.15-1.75, p<.001)	1.02 (0.70-1.47, p=.933)
Tumor number	Single	914 (91.6%)		
	Multiple	84 (8.4%)	2.41 (1.80-3.24, p<.001)	1.08 (0.63-1.86, p=.772)
Macroinvasion	No	895 (89.7%)		
	Yes	103 (10.3%)	1.17 (0.80-1.71, p=.410)	
BCLC	0	97 (9.7%)		
	I	735 (73.6%)	1.20 (0.83-1.75, p=.329)	0.99 (0.66-1.50, p=.980)
	II	61 (6.1%)	2.94 (1.84-4.70, p<.001)	1.25 (0.55-2.84, p=.597)
	III	105 (10.5%)	1.59 (0.97-2.62, p=.066)	0.81 (0.25-2.61, p=.725)
CNLC	Ia	473 (47.4%)		
	Ib	364 (36.5%)	1.64 (1.30-2.07, p<.001)	1.29 (0.86-1.96, p=.222)
	IIa	37 (3.7%)	3.18 (2.07-4.87, p<.001)	1.10 (0.41-2.97, p=.848)
	IIb	9 (0.9%)	4.21 (1.86-9.55, p<.001)	1.80 (0.56-5.74, p=.321)
	IIIa	104 (10.4%)	1.76 (1.20-2.60, p=.004)	1.65 (0.55-5.00, p=.373)
	IIIb	11 (1.1%)	2.31 (1.02-5.25, p=.045)	1.38 (0.48-4.01, p=.548)
Surgical approach	Open	789 (79.1%)		
	Minimal	209 (20.9%)	0.76 (0.57-1.01, p=.058)	
Bleeding	<400	515 (51.6%)		
	≥400	483 (48.4%)	1.55 (1.25-1.91, p<.001)	1.21 (0.97-1.51, p=.093)
Radical resection	Yes	648 (64.9%)		
	No	350 (35.1%)	1.37 (1.11-1.70, p=.003)	1.17 (0.93-1.46, p=.184)
MVI	No	719 (72.0%)		
	Yes	279 (28.0%)	1.63 (1.31-2.03, p<.001)	**1.36 (1.07-1.73, p=.011)**
Pathological grade	Well	76 (7.6%)		
	Moderately	908 (91.0%)	1.38 (0.90-2.11, p=.135)	
	Poorly	14 (1.4%)	1.12 (0.43-2.95, p=.817)	
AFP (ng/ml)	<400	802 (80.4%)		
	≥400	196 (19.6%)	1.22 (0.95-1.58, p=.120)	
Follow TACE	No	891 (89.3%)		
	Yes	107 (10.7%)	8.52 (6.71-10.80, p<.001)	**6.98 (5.41-9.01, p<.001)**
AFP-Size Ratio	Low	499 (50.0%)		
	High	499 (50.0%)	1.41 (1.14-1.74, p=.001)	**1.30 (1.05-1.61, p=.018)**

Abbreviations: BMI, Body mass index; BCLC, Barcelona Clinic Liver Cancer; CNLC, China Clinic Liver Cancer; AFP, alpha-fetoprotein; MVI, microvascular invasion, TACE, transcatheter arterial chemoembolization.

Table S3 Univariate and multivariate Cox regression analyses of the associations between the prognostic factors and the overall survival of the HCC patients after PSM

name	desc	stats	HR (univariable)	HR (multivariable)
Gender	Male	123 (12.3%)		
	Female	875 (87.7%)	1.64 (0.95-2.83, p=.076)	
Age(years)	<60	792 (79.4%)		
	≥60	206 (20.6%)	0.81 (0.55-1.18, p=.272)	
BMI2	<24	630 (63.1%)		
	≥24	368 (36.9%)	0.98 (0.72-1.34, p=.906)	
Cirrhosis	No	554 (55.5%)		
	Yes	444 (44.5%)	1.57 (1.17-2.12, p=.003)	**1.54 (1.13-2.11, p=.007)**
Child-Pugh	A	969 (97.1%)		
	B	29 (2.9%)	1.84 (0.94-3.61, p=.074)	
Hepatitis background	No	155 (15.5%)		
	Hepatitis B	828 (83.0%)	1.17 (0.77-1.79, p=.462)	
	Hepatitis C	15 (1.5%)	0.72 (0.17-3.04, p=.656)	
Tumor size(cm)	<5	544 (54.5%)		
	≥5	454 (45.5%)	1.61 (1.20-2.17, p=.002)	0.94 (0.56-1.58, p=.822)
Tumor number	Single	914 (91.6%)		
	Multiple	84 (8.4%)	1.95 (1.28-2.98, p=.002)	0.68 (0.32-1.44, p=.309)
Macroinvasion	No	895 (89.7%)		
	Yes	103 (10.3%)	2.08 (1.31-3.29, p=.002)	0.23 (0.02-2.31, p=.214)
BCLC	0	97 (9.7%)		
	I	735 (73.6%)	2.15 (1.09-4.23, p=.026)	1.81 (0.88-3.69, p=.105)
	II	61 (6.1%)	4.14 (1.86-9.22, p<.001)	1.40 (0.37-5.26, p=.620)
	III	105 (10.5%)	4.61 (2.11-10.05, p<.001)	3.72 (0.44-31.28, p=.227)
CNLC	Ia	473 (47.4%)		
	Ib	364 (36.5%)	1.72 (1.22-2.42, p=.002)	1.44 (0.81-2.55, p=.211)
	IIa	37 (3.7%)	2.41 (1.24-4.71, p=.010)	1.83 (0.48-7.07, p=.378)
	IIb	9 (0.9%)	5.02 (2.01-12.50, p<.001)	**4.61 (1.09-19.50, p=.038)**
	IIIa	104 (10.4%)	3.26 (2.01-5.30, p<.001)	**4.74 (1.57-14.27, p=.006)**
	IIIb	11 (1.1%)	4.20 (1.68-10.52, p=.002)	**6.33 (1.78-22.58, p=.004)**
Surgical approach	Open	789 (79.1%)		
	Minimal	209 (20.9%)	0.59 (0.37-0.94, p=.027)	0.88 (0.54-1.45, p=.618)
Bleeding	<400	515 (51.6%)		
	≥400	483 (48.4%)	1.91 (1.40-2.60, p<.001)	**1.56 (1.13-2.15, p=.006)**
Radical resection	Yes	648 (64.9%)		
	No	350 (35.1%)	1.62 (1.20-2.18, p=.002)	1.24 (0.90-1.70, p=.194)
MVI	No	719 (72.0%)		
	Yes	279 (28.0%)	1.64 (1.20-2.23, p=.002)	1.11 (0.78-1.57, p=.567)
Pathological grade	Well	76 (7.6%)		
	Moderately	908 (91.0%)	1.43 (0.78-2.63, p=.251)	
	Poorly	14 (1.4%)	0.42 (0.05-3.27, p=.408)	
AFP (ng/ml)	<400	802 (80.4%)		
	≥400	196 (19.6%)	1.34 (0.95-1.91, p=.098)	
Follow TACE	No	891 (89.3%)		
	Yes	107 (10.7%)	4.87 (3.55-6.68, p<.001)	**4.41 (3.12-6.22, p<.001)**
AFP-Size Ratio	Low	499 (50.0%)		
	High	499 (50.0%)	1.65 (1.22-2.24, p=.001)	**1.61 (1.18-2.19, p=.003)**

Abbreviations: BMI, Body mass index; BCLC, Barcelona Clinic Liver Cancer; CNLC, China Clinic Liver Cancer; AFP, alpha-fetoprotein; MVI, microvascular invasion, TACE, transcatheter arterial chemoembolization.
